# There is currently no evidence that high serum leptin and low insulin-like growth factor-1 levels characterise fibromyalgia

**DOI:** 10.1590/1806-9282.20240005

**Published:** 2024-06-17

**Authors:** Josef Finsterer, Fulvio Alexandre Scorza

**Affiliations:** 1 Neurology and Neurophysiology Center – Vienna, Austria.; 2 Universidade Federal de São Paulo/Escola Paulista de Medicinal, Neuroscience Discipline – São Paulo (SP), Brazil.

Dear Editor,

We read with interest Atamer et al.'s article on the serum levels of leptin, growth hormone, insulin-like growth factor-1 (IGF-1), and insulin-like growth factor binding protein-3 in 30 patients with primary fibromyalgia (FM), which were compared with 30 healthy controls^1^. Serum leptin, tender point count, visual analogue scale (VAS) score, FM impact questionnaire score (FMIQS) and Beck depression inventory score (BDIS) were found to be increased and IGF-1 decreased in FM patients^1^. Leptin levels were positively correlated with VAS score, FMIQS, BDIS, tender point count, and disease duration^1^ but negatively correlated with IGF-1^1^. IGF-1 was negatively correlated with age, VAS, FMIQS, BDIS, disease duration, and tender point count^1^. It was concluded that increased serum leptin and decreased IGF-1 levels may be involved in the pathogenesis of FM^1^. The study is impressive, but some points should be discussed.

A limitation of the study is that factors other than FM that influence the level of the parameters analysed in the study were not sufficiently considered and discussed. For example, serum leptin levels depend heavily on the quantity and quality of food intake. High carbohydrate meals increase leptin levels compared to low carbohydrate food. Therefore, we should know what kind of food and how much the included FM patients consumed while starting the 12-h fasting period before blood collection. Serum leptin also increases with the amount of adipose tissue^2^. Therefore, we should know whether all patients had the same amount of body fat and whether leptin levels were correlated with the amount of body fat. Did all patients have the same body mass index? Leptin is an adipokine that regulates appetite and body mass and has many other pleiotropic functions^2^. Leptin acts as a signal to the brain to inhibit food intake and allows excess calories to be stored in fat cells^2^.

In addition to FM, IGF-1 is also low when growth hormone, parathyroid, and oestrogen levels are low. Therefore, we should know whether IGF-1 levels correlated with growth hormone, parathyroid, and oestrogen levels. Again, since high-protein diets can increase IGF-1^3^, it is important to know what type of food the 30 FM patients included consumed before the 12-h fasting period. It is also important to know whether the 30 FM patients consumed a high-fat diet, particularly whether the proportion of unsaturated fat was high. A diet high in unsaturated fatty acids is known to lower IGF-1^3^. Fasting is also known to reduce IGF-1 levels^3^.

Based on these considerations, we do not believe that elevated leptin or low IGF-1 is involved in the pathogenesis of FM. High serum leptin and low IGF-1 are multicausal and, before considering a causal relationship between high leptin/ low IFG-1 and FM, more plausible causes of high leptin/low IGF-1 must be excluded.

In summary, the excellent study has limitations that should be addressed before drawing final conclusions. Clarifying the weaknesses would strengthen the conclusions and could improve the study. Before determining that serum leptin is high and serum IGF-1 is low in FM, all alternative causes of low IGF-1 and high leptin must be considered and ruled out.
